# Proteomic profiling of the serum of patients with COVID‐19 reveals key factors in the path to clinical improvement

**DOI:** 10.1002/ctm2.70201

**Published:** 2025-01-27

**Authors:** Hye Seong, Chae‐Hyeon Lee, Seo‐Gyu Park, Kyoung‐Min Choi, Su‐Min Lee, Jisoo Han, Ha‐Song Bae, Su‐Bhin Han, Sung‐Jin Kim, Eunjung Kim, Jae‐Young Kim, Joon Young Song

**Affiliations:** ^1^ Division of Infectious Diseases, Department of Internal Medicine Korea University Guro Hospital, Korea University College of Medicine Seoul Republic of Korea; ^2^ Asian Pacific Influenza Institute Seoul Republic of Korea; ^3^ Vaccine Innovation Center‐KU Medicine Seoul Republic of Korea; ^4^ Graduate School of Analytical Science and Technology (GRAST) Chungnam National University Daejeon Republic of Korea; ^5^ Natural Product Systems Biology Research Center, Natural Product Informatics Center Korea Institute of Science and Technology Gangneung Republic of Korea

1

Dear Editor,

This study uncovers new molecular insights into the coronavirus disease (COVID‐19) remission process and identifies potential predictive markers through proteomic profiling of patient serum, potentially guiding clinical decision making.

COVID‐19 caused by the SARS‐CoV‐2 virus has triggered a global health crisis. Many proteomic studies using patient sera have been conducted to understand the host's response to this disease and identify potential therapeutic target.^1^, ^2^, ^3^, ^4^ However, the precise mechanisms underlying the diverse clinical presentations of patients with SARS‐CoV‐2 infection remain unclear. Additionally, only a few studies have investigated the molecular factors or biomarkers that may influence clinical improvement of patients who have already exhibited severe symptoms. In this study, proteomic analysis of serum samples from a unique patient cohort, including individuals whose COVID‐19 symptoms worsened and those who improved from moderate or severe conditions, identified 14 differentially expressed proteins (DEPs). Pathway and network analysis of these proteins revealed potential biological processes central to COVID‐19 remission, particularly complement regulation. Remarkably, elevated levels of complement C2 in the patient serum could be an early marker for detecting clinical deterioration in patients with COVID‐19.

The study design, patient clinical data, and the proteomics analysis, including statistical and bioinformatics analysis, are detailed in the Supporting Information (Section 1). Briefly, proteomic analyses were conducted using sera collected from a cohort of 20 patients with COVID‐19 and five healthy individuals as controls. Patients with COVID‐19 were categorised into different prognostic groups based on the National Institute of Allergy and Infectious Disease Ordinal Scale score (Table S1): those who improved from mild COVID‐19 (G1), individuals with deteriorating COVID‐19 symptoms (G2), individuals who improved from moderate to mild COVID‐19 (G3), and those who improved from severe to mild COVID‐19 (G4). Table S2 presents the baseline characteristics of patients with COVID‐19 and healthy controls, and Table S3 presents a comparison of the laboratory test results across the subject groups. After depleting high‐abundance proteins using High‐Select HSA/Immunoglobulin Depletion Resin (Thermo Scientific), sera from patients were processed for proteomic analysis via in‐gel trypsin digestion and liquid chromatography‒mass spectrometry/mass spectrometry (LC‒MS/MS). The study design is illustrated in Figure 1. Using this proteomics approach, a total of 181 proteins were identified and quantified (Figure S1 and Dataset S1). Protein intensities were normalised,^5^ and DEPs (fold‐change > 2, *p* < .05) were identified using MaxQuant software and statistical tools. Pathway enrichment and interaction networks of DEPs were analysed using STRING database and DAVID functional annotation tool.

**FIGURE 1:**
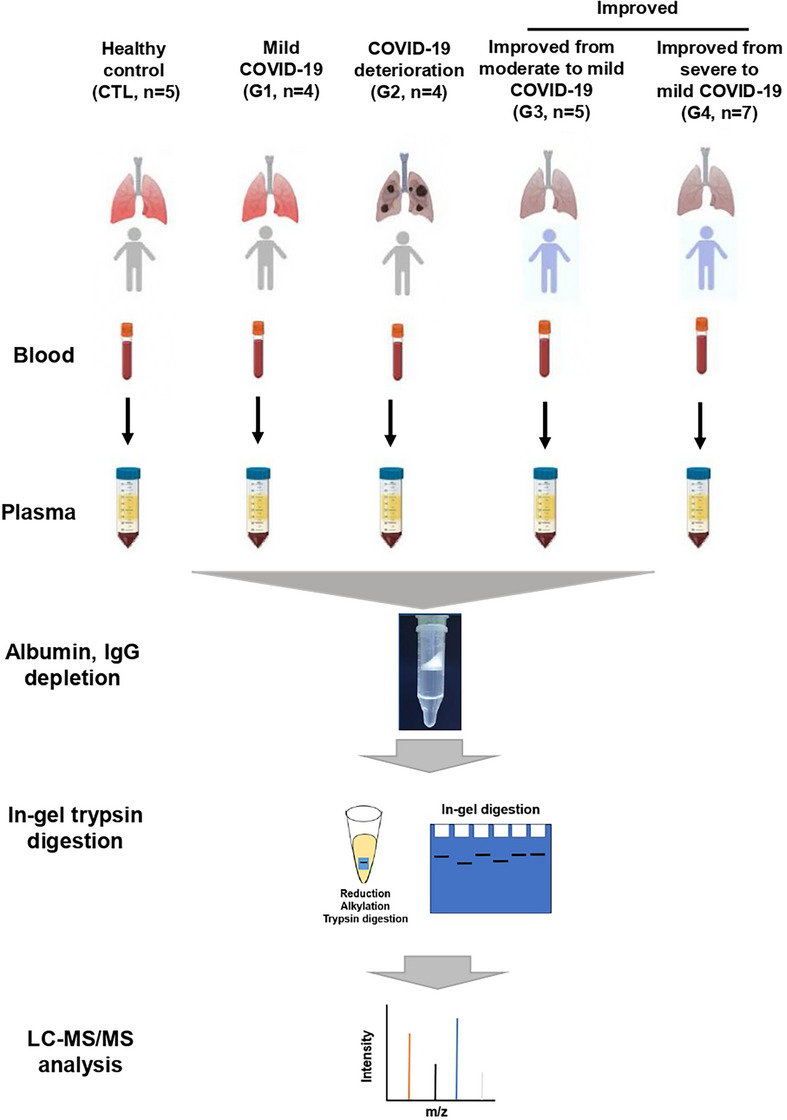
Study design overview. Proteomics analyses were conducted on the serum of four groups of patients with coronavirus disease (COVID‐19) with indicated prognoses and five healthy individuals as the controls. Serum albumin and immunoglobulins were removed, followed by in‐gel digestion. Liquid chromatography‒mass spectrometry (LC‒MS) analysis was performed in technical duplicates, and label‐free quantitation was subsequently conducted.

DEPs were defined based on the criteria of more than a twofold average change and a *p*‐value below.05. Initially, the proteomic differences between healthy controls and group 1 (G1) patients were assessed. This yielded 29 DEPs, with 20 upregulated and nine downregulated in group G1 relative to the healthy controls (Table S4). Visual representations of these findings are shown in Figure S2A‒C. The interaction network from the STRING database emphasised that the interactions were mainly associated with complement activation and acute inflammatory responses (Figure S3A,B and Dataset S2). All patients in groups G2, G3 and G4 underwent the same treatment regimen. G2 patients deteriorated, whereas those in groups G3 and G4 improved to mild conditions. The principal aim of this study was to identify the serum protein markers that differentiate these response categories, potentially providing insight into disease progression and suggesting potential prognostic biomarkers. A comparison between G2 (deteriorating patients) and G3 (improving from moderate to mild) is illustrated in a volcano plot (Figure 2A). Three proteins, namely, PROC, A2MG and CFHA, were identified, with their expression upregulated in the clinically improved group. Conversely, six proteins, CLUS, APOA4, KLKB1, CO2, HV316 and PON1, were less abundant in the clinically improved group (Figure 2B,C and Dataset S3).

**FIGURE 2:**
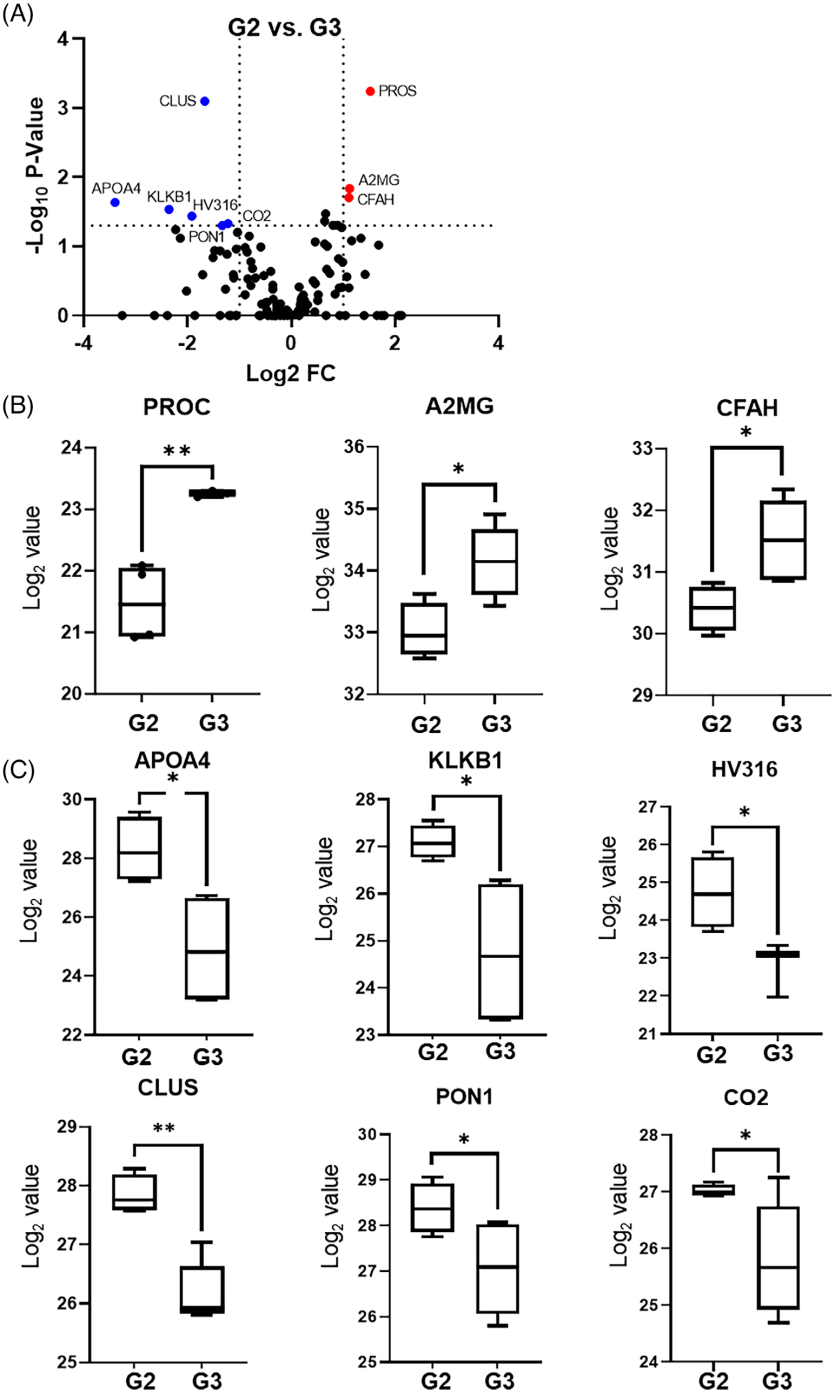
Comparison between deteriorating (G2) and improved from moderate coronavirus disease (COVID‐19) (G3) groups. (A) Volcano plot showing differentially expressed proteins (DEPs) between G2 and G3. Nonaxial vertical and horizontal lines denote twofold change and *p* < .05, respectively. (B and C) Box plots for upregulated DEPs (B) and downregulated DEPs (C) in the improved group (G3). ^*^ *p* < .05 and ^**^ *p* < .01.

A parallel comparison between G2 and G4 resulted in the identification of three upregulated (C4BPA, CHLE and TTHY) and three downregulated (FETUB, CO2 and KAIN) proteins in G4 (Figure 3A‒C and Dataset S4). Notably, the CO2 (or complement C2) levels were consistently lower in both clinically improved groups. Given its role in the complement system and previous research linking complement dysregulation with severe COVID‐19 outcomes,^6^, ^7^, ^8^ higher basal CO2 levels may be associated with unfavorable patient outcomes. We have identified CO2, along with C4BPA, CFAH, KLKB1, PROC and A2MG, as notable biomarkers for determining COVID‐19 prognosis.

**FIGURE 3:**
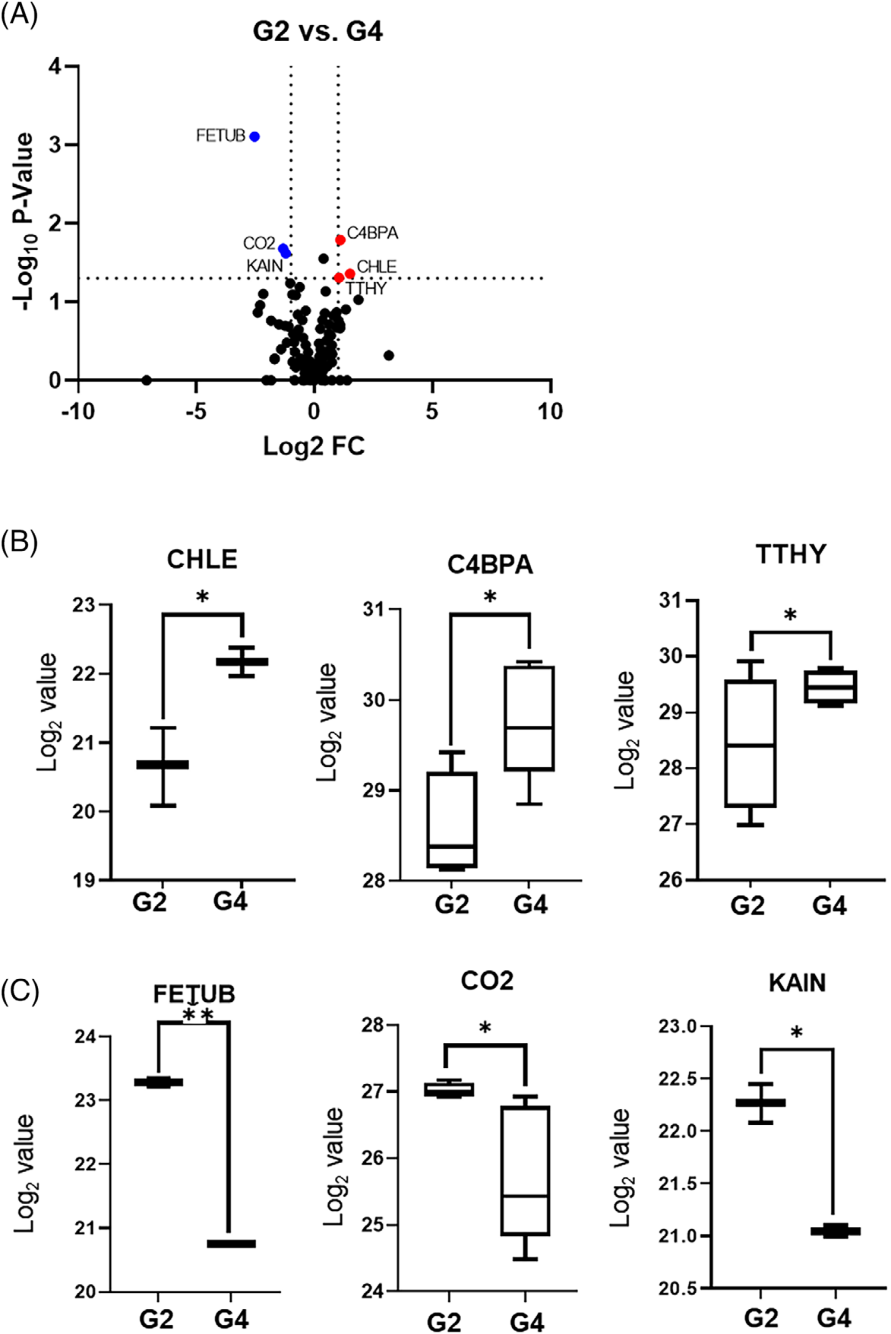
Comparison between deteriorating (G2) and improved from severe coronavirus disease (COVID‐19) (G4) groups. (A) Volcano plot showing differentially expressed proteins (DEPs) between G2 and G4. Nonaxial vertical and horizontal lines denote twofold change and *p* < .05, respectively. (B and C) Box plots for upregulated DEPs (B) and downregulated DEPs (C) in the improved group (G4). ^*^ *p* < .05 and ^**^ *p* < .01.

Table S5 summarises 14 DEPs (six upregulated and eight downregulated) identified in the clinically improved groups from our two comparisons. The STRING database was used to map these proteins, revealing extensive interrelations, except for FETUB (Figure 4A). This relationship suggests the potential involvement of shared biological pathways or processes. Notably, functional annotation of these DEPs revealed a strong association with complement activation pathways (Figure 4B). Further analyses employing the DAVID functional annotation tool showed similar results, with persistent enrichment in complement pathways and related functionalities, such as innate immunity and blood coagulation (Figure 4C). Finally, the KEGG pathway analysis identified DEPs intricately tied to the complement and coagulation cascades (Figure 4D).

**FIGURE 4:**
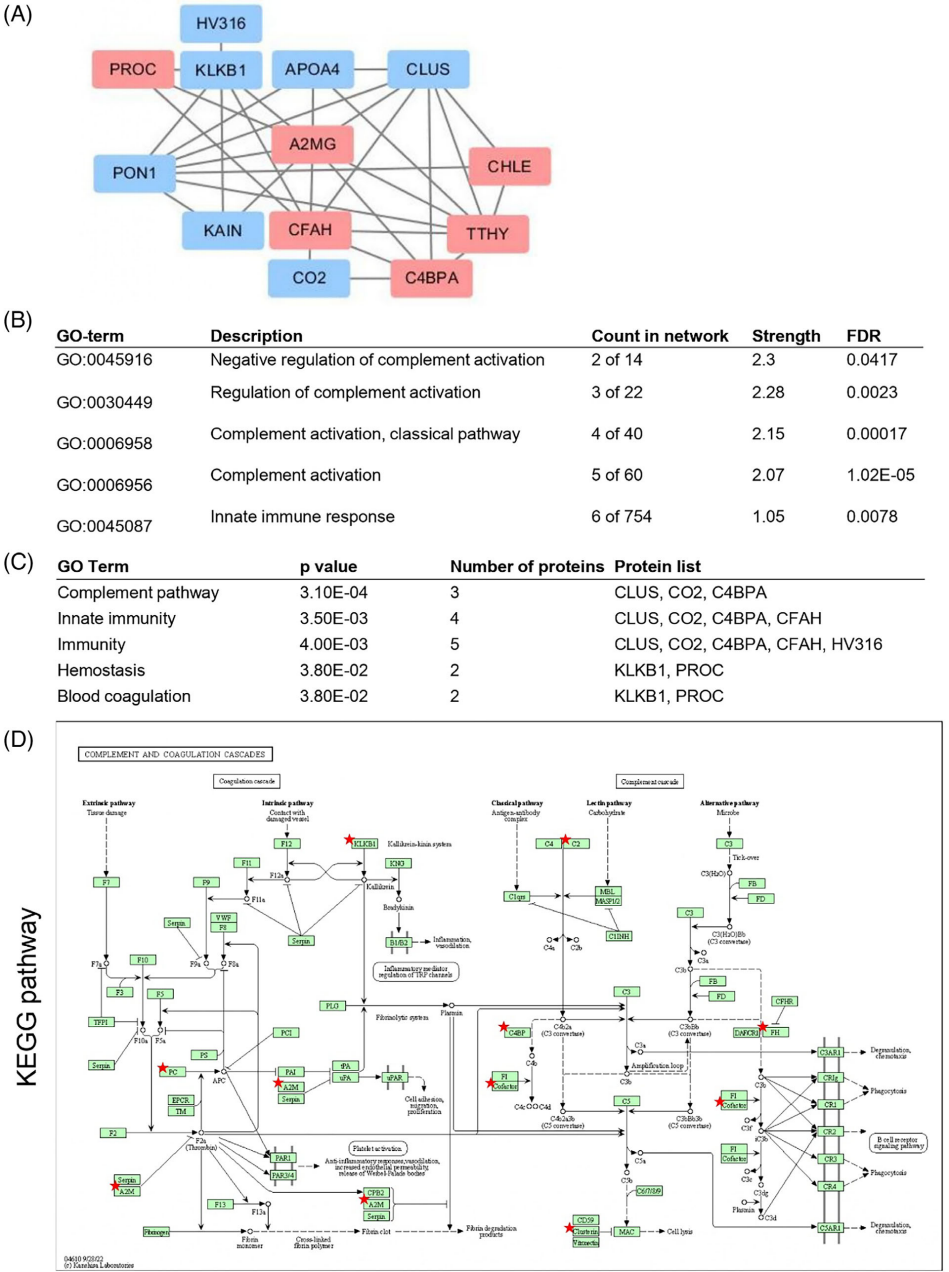
Biological pathways and protein‒protein interaction network involved in coronavirus disease (COVID‐19) remission. (A) Protein‒protein interaction network for the 14 differentially expressed proteins (DEPs) in the improved group. Red indicates an increase, while blue indicates a decrease in the improved group. (B and C) Biological pathways enriched in the DEPs based on the STRING (B) and DAVID (C) databases. (D) DEPs in the complement and coagulation cascades are denoted by a red star.

This study highlights key proteomic mechanisms influencing COVID‐19 outcomes. Complement activation, particularly CO2, emerged as a major contributor to disease severity, driving inflammatory responses and coagulopathy. Regulatory proteins such as C4BPA and CFAH, upregulated in improved patients, could play protective roles by mitigating complement‐induced tissue damage. Altered levels of KLKB1, PROC and A2MG link vascular dysfunction and thrombogenesis to clinical improvement of COVID‐19, underscoring the interplay between inflammation, coagulation and vascular integrity. These findings provide deeper insights into COVID‐19 pathophysiology, suggesting that therapeutic targeting of complement and coagulation pathways could improve clinical outcomes and aid clinical improvement in severely affected patients. Detailed evidence and explanations for our findings are provided in the Supporting Information (Section 2).

In conclusion, the proteomic investigation of the sera of patients with COVID‐19 with varying prognoses revealed a panel of proteins that are potentially essential for disease progression and response to treatment. With the complement system emerging as a central player, this study not only offers a deeper molecular understanding of the disease but also highlights potential therapeutic avenues and prognostic markers warranting further exploration.

## AUTHOR CONTRIBUTIONS

*Conception and design*: Hye Seong, Jae‐Young Kim and Joon Young Song. *Acquisition of data*: Seo‐Gyu Park, Kyoung‐Min Choi, Su‐Min Lee, Jisoo Han, Ha‐Song Bae, Su‐Bhin Han, Sung‐Jin Kim and Eunjung Kim. *Analysis and interpretation of data*: Hye Seong, Jae‐Young Kim and Joon Young Song. *Writing and reviewing the manuscript*: Hye Seong, Chae‐Hyeon Lee, Jae‐Young Kim and Joon Young Song. All the authors have read and agreed to the published version of the manuscript.

## CONFLICT OF INTEREST STATEMENT

The authors declare they have no conflicts of interest.

## ETHIS STATEMENT

This study was approved by the Institutional Review Board of Korea University Guro Hospital (2020GR0570), and written informed consent was obtained from all participants. All procedures were performed according to the ethical standards of the institutional and/or national research committee and in accordance with the Declaration of Helsinki.

## Supporting information



Supporting Information

Supporting Information

## Data Availability

Data that support the findings of this study are presented in the main article and Supporting Information files. Further data that support the findings of this study are available upon request.

## References

[1] Shen B , Yi X , Sun Y , et al. Proteomic and metabolomic characterization of COVID‐19 patient sera. Cell. 2020;182(1):59‐72. e15. 32492406 10.1016/j.cell.2020.05.032 PMC7254001

[2] Park J , Kim H , Kim SY , et al. In‐depth blood proteome profiling analysis revealed distinct functional characteristics of plasma proteins between severe and non‐severe COVID‐19 patients. Sci Rep. 2020;10(1):22418. 33376242 10.1038/s41598-020-80120-8 PMC7772338

[3] Zhong W , Altay O , Arif M , et al. Next generation plasma proteome profiling of COVID‐19 patients with mild to moderate symptoms. EBioMedicine. 2021;74:103423. 10.1016/j.ebiom.2021.103723 PMC8626206 34844191

[4] D'Alessandro A , Thomas T , Dzieciatkowska M , et al. Serum proteomics in COVID‐19 patients: altered coagulation and complement status as a function of IL‐6 level. J Proteome Res. 2020;19(11):4417‐4427. 32786691 10.1021/acs.jproteome.0c00365 PMC7640953

[5] Chawade A , Alexandersson E , Levander F . Normalyzer: a tool for rapid evaluation of normalization methods for omics data sets. J Proteome Res. 2014;13(6):3114‐3120. 24766612 10.1021/pr401264n PMC4053077

[6] Yu J , Gerber GF , Chen H , et al. Complement dysregulation is associated with severe COVID‐19 illness. Haematologica. 2021;107(5):1095. 10.3324/haematol.2021.279155 PMC9052917 34289657

[7] Java A , Apicelli AJ , Liszewski MK , et al. The complement system in COVID‐19: friend and foe? JCI Insight. 2020;5(15):e140711. 32554923 10.1172/jci.insight.140711 PMC7455060

[8] Kim AHJ , Wu X , Atkinson JP . The beneficial and pathogenic roles of complement in COVID‐19. Cleve Clin J Med. 2020. 10.3949/ccjm.87a.ccc065 PMC8079550 33115882

