# Supplementary material for: Proteomic profiling of the serum of patients with COVID‐19 reveals key factors in the path to clinical improvement
Source: Clin Transl Med. 2025 Jan 27;15(2):e70201. doi: 10.1002/ctm2.70201 (PMC11772101; doi:10.1002/ctm2.70201)
Supplement: Supplementary file 1 — Supporting Information [file CTM2-15-e70201-s002.docx]

**Supplementary Materials to: Proteomic Profiling of the Serum of Patients with COVID-19 Reveals Key Factors in the Path to Clinical Improvement**

This Appendix provides further methodological details and additional Tables and Figures for “Proteomic Profiling of the Serum of Patients with COVID-19 Reveals Key Factors in the Path to Clinical Improvement”

**Contents**

**Section 1: Supplementary materials and methods 2**

Study Design and Participants 2

Sample Collection 2

Statistical Analysis of Clinical Data 2

Proteomic Analysis 2

Statistical and Bioinformatic Analysis of the Proteomics Data 3

**Section 2: Supplementary discussion 3**

**Section 3: Supplementary figures 6**

Figure S1. Hierarchical clustering and heat map of protein intensities 6

Figure S2. Comparison between mild COVID-19 (G1) and the healthy control group 8

Figure S3. Biological pathways and protein-protein interaction network involved in COVID-19 infection 10

**Section 4: Supplementary tables 11**

Table S1. National Institute of Allergy and Infectious Disease Ordinal Scale (NIAID-OS) 11

Table S2. Baseline characteristics of COVID-19 case and healthy control 12

Table S3. Comparison of laboratory test results of subjects 14

Table S4. Differentially expressed proteins between mild case group (G1) and healthy control group 16

Table S5. Differentially expressed proteins between deterioration (G2) and improved patient groups (G3 and G4) 18

**Section 5: References 19**

**Section 1: Supplementary materials and methods**

**Materials and methods**

**Study Design and Participants**

From January 8 to September 9, 2021, a prospective cohort study was conducted at two tertiary hospitals in the Republic of Korea—Korea University Guro Hospital and Chungbuk National University Hospital. We enrolled 25 individuals, including 20 patients with COVID-19 and five healthy individuals as the control.

COVID-19 diagnosis was confirmed using real-time (RT) polymerase chain reaction (RT-PCR) of nasopharyngeal specimens. Patients who had taken probiotics within four weeks prior to presenting with symptoms were excluded from the study. Healthy controls were chosen after ensuring that they had not taken medications, such as antibiotics, probiotics, laxatives, and motility drugs, within the four weeks leading up to the study, as such medications could influence the microbiome. All controls tested negative for SARS-CoV-2 by both nasopharyngeal RT-PCR and serological tests. Detailed patient data, including demographics, medications, radiographic findings, laboratory results, and dietary records, were compiled by two trained physicians.

To further refine our analysis, patients were classified based on the National Institute of Allergy and Infectious Disease Ordinal Scale score (NIAID-OS; Supplementary Table 1) into four categories:

- G1: Patients who improved from mild COVID-19 and maintained a NIAID-OS of 4 (day 0) during their hospital stay.
- G2: Patients who experienced deterioration, worsening from an NIAID-OS of 4 or 5 (day 0) to a score of 7 or 8.
- G3: Patients who showed improvement from moderate to mild COVID-19, transitioning from an NIAID-OS of 5 (day 0) to 4.
- G4: Patients who improved from severe to mild COVID-19, from an NIAID-OS of 6 (day 0) to 4.

We analyzed the proteins from day 0 samples to identify early prognostic markers that could predict COVID-19 progression.

**Sample Collection**

Blood samples were collected on enrollment (day 0), then placed in a serum-separating tube and centrifuged at 2500 rpm at 4 °C for 10 minutes. Following this, the serum-containing supernatant was transferred into a clean plastic screw-cap vial and stored at −80 °C for future analysis.

**Statistical Analysis of Clinical Data**

Continuous variables are presented as median ± interquartile range, while categorical data are shown as numbers (percentages). To analyze the differences among the four prognostic groups, the Kruskal–Wallis test was applied with Bonferroni correction for multiple comparisons. All statistical analyses were performed using R (version 4.1.2), adjusting the p-values for multiple tests. Statistical significance was set at a p-value of 0.05.

**Proteomic Analysis**

To enhance the identification of low-abundance proteins, serum samples were depleted of high-abundance proteins using High-Select HSA/Immunoglobulin Depletion Resin (Thermo Scientific) following the manufacturer’s instructions. Subsequently, aliquots corresponding to 15 μg of protein from each sample were subjected to in-gel digestion as described in our previous study[1]. The resulting tryptic peptides were analyzed using LC-MS (Korea Basic Science Institute, Ochang, Republic of Korea), and the MS and MS/MS spectra were analyzed using the MaxQuant software (ver. 1.5.3.8) as previously described[2, 3]. Briefly, chemical contaminants in the tryptic peptide mixture were removed using an MGU30-18 trapping column (LC Packings). The cleaned peptides were then transferred to a 10 cm × 75 μm IDC18 reverse phase column (PROXEON, Odense, Denmark) at a flow rate of 300 nL/min. The peptides were separated with a gradient of 0–65% acetonitrile over an 80-minute period. Tandem mass spectrometry (MS/MS) was performed using a Q-Exactive Plus mass spectrometer (Thermo Scientific, Waltham, MA, USA) operating in data-dependent mode. Each full MS scan (m/z range 400–2000) was followed by three MS/MS scans targeting the most abundant precursor ions identified in the mass spectrum. The RAW proteomics data have been deposited on MassIVE database with accession number MSV000094722 (https://doi.org/10.25345/C5445HQ0N). FTP for reviewers : ftp://MSV000094722@massive.ucsd.edu, Password: cancer. The search parameters for MaxQuant analysis were primarily set to the default values provided by MaxQuant, which are optimized for high accuracy and reproducibility across a wide range of proteomic experiments.

**Statistical and Bioinformatic Analysis of the Proteomics Data**

Protein intensities across the samples were normalized using the Normalyzer tool (<https://normalyzerde.immunoprot.lth.se/>; [4] ). Each sample was subjected to two technical LC-MS analyses. If data from one of the technical replicates were missing, the values from the remaining replicates were used. Proteins undetected in both technical replicates were considered as "undetected." Mean values and p-values were computed using Excel (Microsoft), and volcano and box plots were created using GraphPad Prism. Differentially expressed proteins (DEPs) were defined as those with an average fold-change greater than two-fold and a p-value less than 0.05. The interaction networks for these DEPs were explored using the STRING database (<https://string-db.org>) and visualized using Cytoscape (<http://cytoscape.org>). Pathway enrichment analysis was conducted using the STRING database and DAVID functional annotation tool (<https://david.ncifcrf.gov/tools.jsp>). For hierarchical clustering, the normalized protein list was clustered using an unsupervised hierarchical method with an Euclidian distance function (R script), as described previously[3].

**Section 2: Supplementary discussion**

**Discussion**

Numerous proteomic investigations have discerned the differences between severe and non-severe COVID-19 manifestations; however, a gap remains in the understanding of the molecular determinants that determine clinical improvement in severely affected patients. This study aimed to bridge this gap by discerning the proteomic differences between these two distinct patient groups. It recruited. patients who initially presented with moderate-to-mild COVID-19, comparing those who deteriorated to severe disease with those that improved without worsening thereafter. The extensive interconnectivity observed among the identified DEPs highlights complement activation and the innate immune response (Figures 4B and C), two processes that are deeply entwined and implicated in COVID-19 progression. Beyond the direct effects of the virus, exacerbated complement activation can induce collateral tissue damage, contributing to severe manifestations of the disease[5]. These results provide additional support for this hypothesis, suggesting that the complement pathway may be crucial in determining the clinical improvement outcome of patients with COVID-19.

Notably, CO2 (Complement C2) was consistently upregulated in the patients with deteriorating symptoms in both the comparative analyses. Complementary systems are an area of significant interest in the context of COVID-19. Complement activation is intrinsically linked to inflammation and coagulation, both of which are pertinent to deteriorating COVID-19 conditions[6]. Previous studies have shown that dysregulation of the complement system is associated with severe SARS-CoV-2 infection, often implicating it in cascading inflammatory responses and multi-organ damage[5-7]. Complement components, particularly C3 and C5, have been previously reported to be elevated in severe COVID-19 cases, and are linked with negative outcomes[8-10]. In the classical pathway, CO2 plays a pivotal role in the formation of C3 convertase, which is central to the complement activation process[11]. Given that complement C2 functions as a key factor in the early activation phase of the classical pathway, these results suggest that CO2 is a sensitive marker for clinical deterioration in COVID-19.

Consistent with these observations, increased expression of proteins associated with complement regulation and tissue recovery from damage caused by aberrant complement activation was observed in the improved group. Specifically, the expression of the C4b-binding protein alpha chain (C4BPA) was upregulated in the improved group (G4) compared to that in the deteriorated group (G2; Figure 3B). C4b-binding protein alpha chain (C4BPA) is a major regulatory protein of the classical pathway of the complement system and plays a critical role in controlling complement activation by binding to C4b and promoting the decay of C3 and C5 convertases, thus inhibiting the progression of the complement cascade[12]. Additionally, complement factor H-related protein 1 (CFAH), whose expression was upregulated in G3 compared to G2 (Figure 2B), is involved in regulating the alternative pathway of the complement system by disrupting convertase enzymes. These proteins play crucial roles in protecting host tissues from the unintended damage caused by complement activation[13]. Taken together, these results indicate an association between these proteins and the improvement process, emphasizing the conceivable influence of complement components in shaping disease prognosis.

In this study, alongside CO2, C4BPA, and CFAH, three additional proteins, KLKB1, PROC, and A2MG, have been identified as notable biomarkers in determining COVID-19 prognosis. These proteins are intricately linked to vascular function and thrombus formation in the acute phase and can influence long-term outcomes by modulating the risk of cardiovascular complications. KLKB1 is instrumental in the activation of factor XII and pre-kallikrein, thereby initiating bradykinin production. This process is essential for triggering the intrinsic coagulation pathway and for releasing bradykinin, a critical inflammatory mediator that enhances vascular permeability and induces vasodilation[14]. Under normal physiological conditions, KLKB1 and other plasma kallikreins act as cardioprotective agents. However, dysregulation of these genes is associated with an increased risk of cardiovascular pathology[15]. This study suggests that elevated KLKB1 levels in patients with COVID-19 could exacerbate the risk of thrombogenesis, potentially leading to critical complications. PROC, a serine protease synthesized in the liver, plays a pivotal role in hemostasis. It is activated by thrombin-bound thrombomodulin in endothelial cells, which converts it to its active form, protein C. Activated protein C deactivates the coagulation factors Va and VIIIa, effectively modulating the coagulation process to prevent hypercoagulability[16]. Notably, Shu et al. (2020) demonstrated that severe COVID-19 cases are often characterized by diminished PROC levels compared with milder cases[17]. The protective role of activated protein C, particularly in safeguarding endothelial cells and maintaining vascular integrity, underscores its significance[18]. A reduction in PROC levels in COVID-19 patients may be a critical factor in the exacerbation of vascular damage and coagulopathy. A2MG is a crucial regulator of coagulation, fibrinolysis, and inflammation as it inhibits a spectrum of proteases, including thrombin, plasmin, and trypsin[19]. In COVID-19, reduced A2MG levels may lead to uncontrolled protease activity, contributing to inflammatory responses, thrombus development, and other pathological changes, ultimately worsening prognosis.

This study has several limitations. First, the sample size was relatively modest. The sample sizes were determined based on initial feasibility and the exploratory nature of our research, which aims to identify preliminary biomarkers for further investigation. Additionally, the small sample size resulted from stringent inclusion and exclusion criteria designed to minimize confounding factors, ensuring the reliability and specificity of our findings. Specifically, our inclusion criteria required participants to be enrolled within 48 hours of symptom onset or worsening. Furthermore, we excluded individuals who had used immunosuppressants, probiotics, antibiotics, laxatives, or motility drugs within the previous month, as these could affect immunogenicity and the gut microbiota. We also excluded individuals with unstable underlying medical conditions before COVID-19 development. These strict criteria were essential to our study's goal of identifying clear and meaningful biomarkers. Despite the small sample size, our study employed rigorous statistical methods to ensure that the results are robust and provide foundational insights for larger follow-up studies. Second, this study focused on serum protein profiles, which, while offering invaluable insights into systemic responses, may not encapsulate localized tissue responses or specific cellular interactions with the virus. Integrating proteomics with transcriptomic or metabolomic data could provide a more comprehensive view of host-pathogen interactions. Finally, we could not exclude the potential impact of pre-existing medical conditions on protein expression in this study. To minimize the potential confounding effects, we restricted our study population to patients whose underlying medical conditions were stable prior to their COVID-19 diagnosis. Because we did not perform direct comparisons of protein expression between patients with and without prior medical conditions, we recognize the importance of considering this factor in the interpretation of our results. Proteins like CLUS, which are known to have non-specific functions such as roles in apoptosis and post-myocardial infarction recovery, may indeed be influenced by underlying conditions. Additional studies are needed to validate these findings in larger cohorts and further investigate the specificity of these proteins to COVID-19.

In conclusion, the proteomic investigation of the sera of patients with COVID-19 with varying prognoses revealed a panel of proteins that are potentially essential for disease progression and response to treatment. With the complement system emerging as a central player, this study not only offers a deeper molecular understanding of the disease but also highlights potential therapeutic avenues and prognostic markers warranting further exploration.

**Section 3: Supplementary figures**


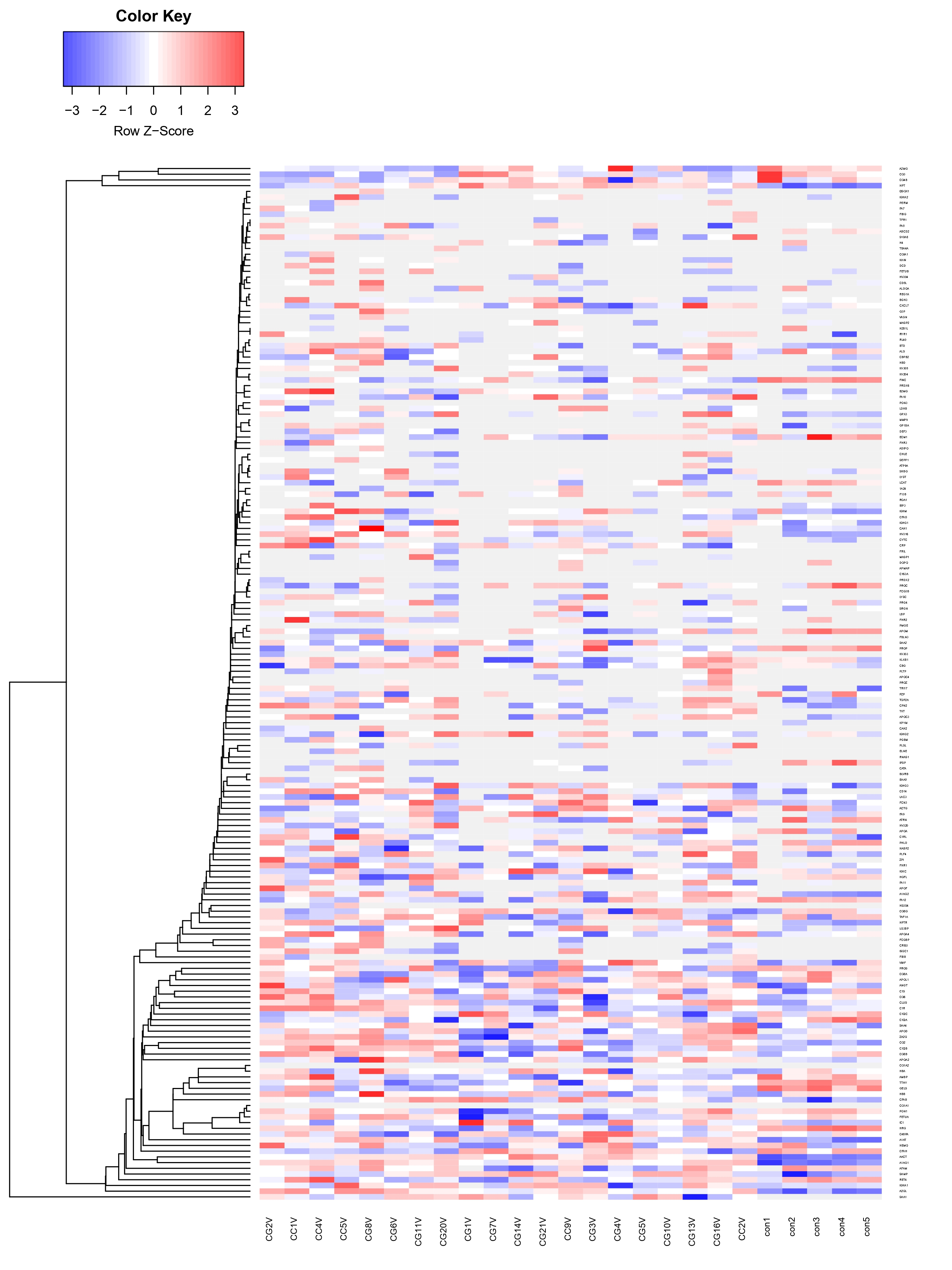


G1

G2

G3

G4

control

**Figure S1. Hierarchical clustering and heat map of protein intensities.** Protein intensity changes from the baseline intensity were clustered using an unbiased hierarchical method with an Euclidian distance function, resulting in a few different clusters. Rows represent proteins, and columns represent patient IDs. Red indicates an increase in intensity, and blue indicates a decrease.


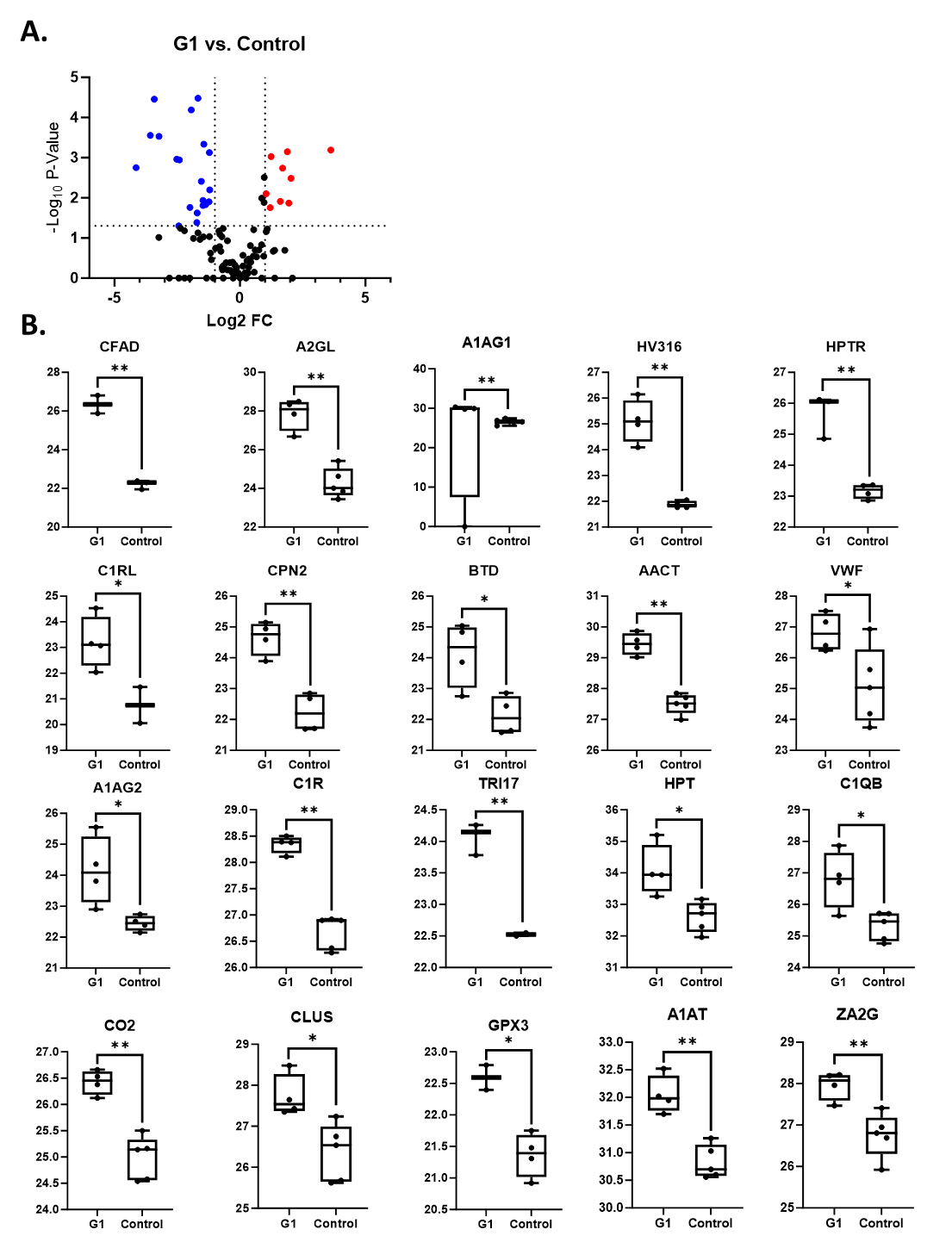


**
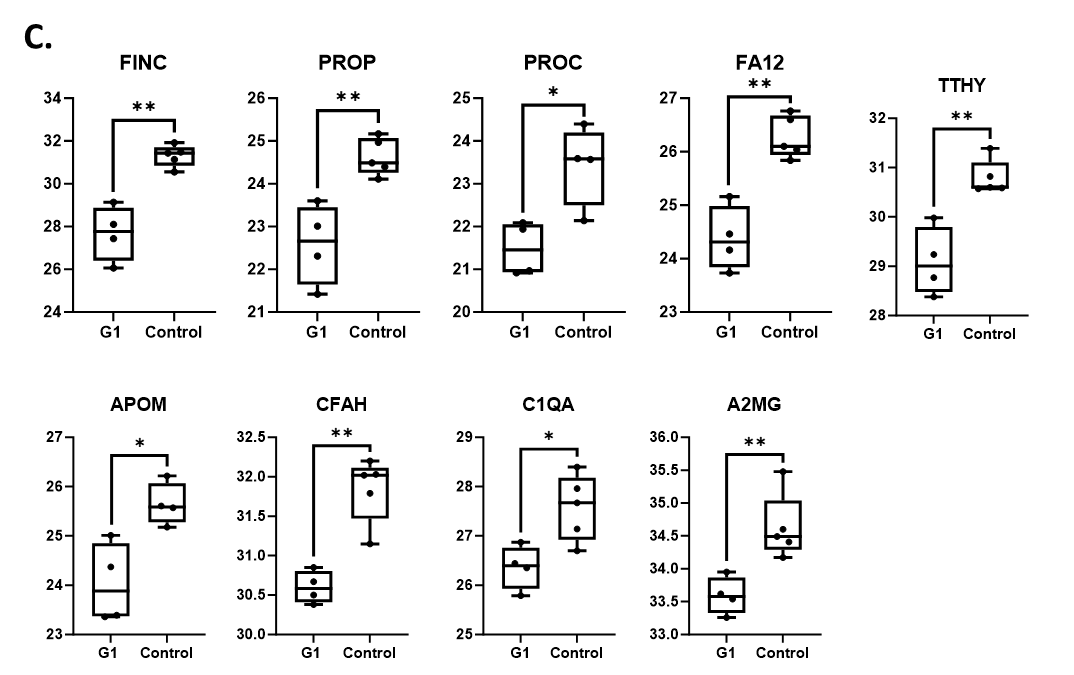
**

**Figure S2. Comparison between mild COVID-19 (G1) and the healthy control group.** A. Volcano plot showing differentially expressed proteins (DEPs) between G1 and the healthy control group. Nonaxial vertical and horizontal lines denote two-fold change and p = 0.05, respectively. B and C. Box plots for DEPs upregulated (B) and downregulated (C) in G1.

**
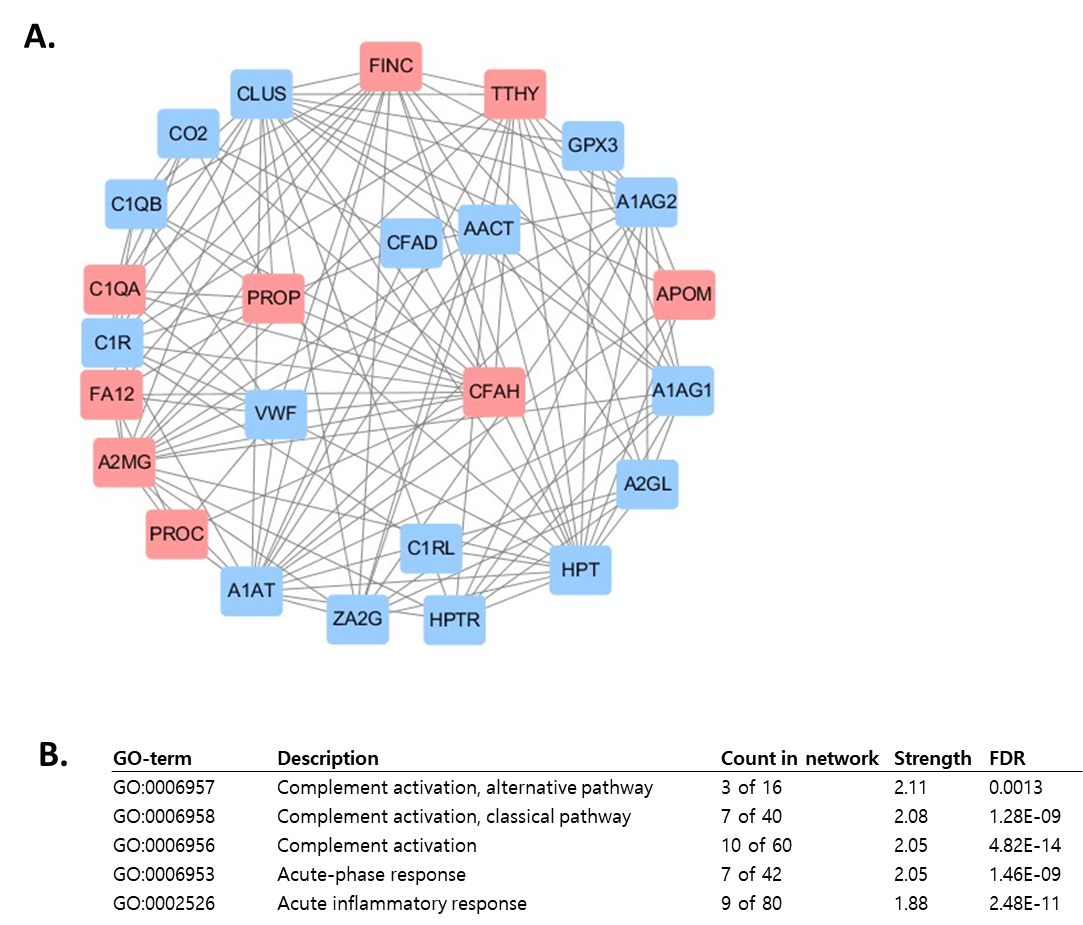
**

**Figure S3. Biological pathways and protein-protein interaction network involved in COVID-19 infection.** A. Protein-protein interaction network for 29 differentially expressed proteins (DEPs) indicated in Figures S1B and S1C. Red indicates an increase in the control group, and blue indicates a decrease. B. Biological pathways enriched in the DEPs from the STRING database.

**Section 4: Supplementary tables**

| **Table S1. National Institute of Allergy and Infectious Disease Ordinal Scale (NIAID-OS)** | | |
| --- | --- | --- |
| **Patients** | **NIAID-OS** | **Description** |
|  | **1** | Not hospitalized, no limitations on activities |
|  | **2** | Not hospitalized, no limitations on activities and/or requiring home oxygen |
|  | **3** | Hospitalized, not requiring supplemental oxygen- no longer requires ongoing medical care |
| Population enrolled | **4** | Hospitalized, nor requiring supplemental oxygen- requiring ongoing medical care |
|  | **5** | Hospitalized, requiring supplemental oxygen |
|  | **6** | Hospitalized, on non-invasive ventilation or high flow oxygen devices |
|  | **7** | Hospitalized, on mechanical ventilation or ECMO |
|  | **8** | Death |
| ECMO, extracorporeal membrane oxygenation. | | |

| **Table S2. Baseline characteristics of COVID-19 case and healthy control** | | | | | | |
| --- | --- | --- | --- | --- | --- | --- |
| **Characteristics** | **COVID-19 case (*n* = 20)** | | | | ***P* value** | **Healthy control**  **(*n* = 5)** |
|  | **Group 1 (*n* = 4)** | **Group 2 (*n* = 4)** | **Group 3 (*n* = 5)** | **Group 4 (*n* = 7)** |  |  |
| **Age (years)** | 57.5±31.0 | 59.0±35.0 | 67.0±21.0 | 68.0±22.0 | 0.698 | 45.0±31.0 |
| **Male sex (%)** | 1 (25.0) | 3 (75.0) | 3 (60.0) | 4 (57.1) | 0.642 | 2 (40.0) |
| **BMI (kg/m^2^)** | 20.5±13.3 | 25.5±8.6 | 24.0±5.5 | 24.1±5.2 | 0.677 | 25.9±8.3 |
| **Duration from symptom onset to enrollment (days)** | 3.5±7 | 4.5±4 | 10.0±5 | 6.0±8 | 0.071 | - |
| **ATB use within 1week. yes (%)** | 1 (25.0) | 2 (50.0) | 3 (60.0) | 5 (71.4) | 0.542 | - |
| **Duration of total ATB use (days)** | 7.5±17^ab^ | 24.0±17^a^ | 9.0±13^b^ | 10.0±7^ab^ | 0.021 | - |
| **Dexamethasone use, yes (%)** | 0 (0.0) | 4 (100.0) | 5 (100.0) | 7 (100.0) | <0.001 | - |
| **Remdesivir use, yes (%)** | 0 (0.0) | 3 (75.0) | 3 (60.0) | 7 (100.0) | 0.006 | - |
| **Pneumonia, yes (%)** | 2 (50.0) | 4 (100.0) | 5 (100.0) | 7 (100.0) | 0.063 | - |
| **C_t_ value, RdRp gene** | 20.23±17.5 | 21.55±7.72 | 30.12±6.55 | 25.73±7.22 | 0.113 | - |
| **Charlson comorbidity index** | 4.7±3.5 | 2.0±5.0 | 2.7±2.6 | 4.0±4.4 | 0.814 | 0.5±2.4 |
| **Underlying diseases** |  |  |  |  |  |  |
| Hypertension | 3 (75.0) | 2 (50.0) | 1 (20.0) | 3 (42.9) | 0.482 | - |
| Myocardial infarction | 0 (0.0) | 0 (0.0) | 0 (0.0) | 1 (14.3) | >0.999 | - |
| Cerebrovascular attack | 0 (0.0) | 1 (25.0) | 0 (0.0) | 0 (0.0) | 0.400 | - |
| Dementia | 1 (25.0) | 1 (25.0) | 0 (0.0) | 0 (0.0) | 0.200 | - |
| Diabetes mellitus | 2 (50.0) | 2 (50.0) | 1 (20.0) | 3 (42.9) | 0.807 | - |
| Hemiplegia | 0 (0.0) | 1 (25.0) | 0 (0.0) | 0 (0.0) | 0.400 | - |
| End-stage renal disease | 2 (50.0) | 0 (0.0) | 0 (0.0) | 0 (0.0) | 0.063 | - |
| Group 1, improved from mild COVID-19; Group 2, deterioration; Group 3, improved from moderate to mild severity; Group 4, improved from severe to mild severity.  BMI, body mass index; ATB, antibiotics; C_t_, cycle threshold; RdRp gene, RNA-dependant RNA polymerase gene.  *Superscripts* (^a,b^). For a particular variable, mode means with different superscript are significantly (*p* <0.05) different. Mode means with same superscripts are not significantly (*p* >0.05) different. When only one contrast is significant, one of the cells means has no superscript attached. The pair of cell means that is significant has different superscripts. | | | | | | |

Continuous variables are shown as median ± interquartile range (IQR) and categorical variables as numbers (percentage).

| **Table S3. Comparison of laboratory test results of subjects** | | | | | | |
| --- | --- | --- | --- | --- | --- | --- |
| **Characteristics** | **COVID-19 cases (*n* = 20)** | | | | ***P* value** | **Healthy control**  **(*n* = 5)** |
|  | **Group 1 (*n* = 4)** | **Group 2 (*n* = 4)** | **Group 3 (*n* = 5)** | **Group 4 (*n* = 7)** |  |  |
| WBC (10^3^cells/uL) | 5.74±10.88 | 6.95±4.95 | 4.45±3.30 | 6.30±5.64 | 0.563 | 4.60±2.85 |
| Neutrophil (cells/uL) | 3926.96±8107.89 | 6002.80±4475.68 | 3524.40±3145.90 | 4794.30±7026.50 | 0.712 | 2196.40±2004.60 |
| Lymphocyte (cells/uL) | 1195.72±2408.66 | 336.50±360.43 | 449.45±422.45 | 648.90±813.90 | 0.136 | 1378.80±1127.7 |
| Monocyte (cells/uL) | 559.44±800.26 | 266.50±485.37 | 231.00±369.15 | 435.00±668.50 | 0.397 | 439.20±235.5 |
| Hemoglobin (g/dL) | 12.5±1.8 | 13.1±5.0 | 14.2±1.6 | 12.2±3.0 | 0.441 | 12.8±3.1 |
| MCV (fL) | 93.9±11.0 | 94.1±5.1 | 90.7±3.2 | 92.1±6.6 | 0.530 | 95.0±7.3 |
| MCH (pg) | 30.5±2.7 | 31.7±2.9 | 30.2±1.4 | 31.1±2.8 | 0.616 | 31.0±3.1 |
| MCHC (g/dL) | 32.5±0.9 | 33.5±1.5 | 33.9±1.0 | 33.7±1.2 | 0.092 | 33.3±1.4 |
| Platelet count (10^3^/uL) | 154.5±185.0 | 165.0±75.0 | 205.0±165.0 | 189.0±124.0 | 0.604 | 252.0±122.0 |
| BUN (mg/dL) | 47.5±43.0 | 17.5±10.7 | 18.2±13.2 | 16.6±2.7 | 0.498 | 15.5±4.8 |
| Creatinine (mg/dL) | 3.89±8.31 | 0.62±0.26 | 0.65±0.31 | 0.75±0.41 | 0.357 | 0.69±0.35 |
| Total protein (g/dL) | 6.1±0.8 | 6.7±1.6 | 6.4±1.5 | 6.6±0.7 | 0.590 | 7.4±0.0 |
| Albumin (g/dL) | 3.5±1.1 | 3.4±0.7 | 3.5±0.5 | 3.5±0.7 | 0.470 | 4.1±0.4 |
| AST (IU/L) | 21.0±58.0 | 38.0±15.0 | 45.0±65.0 | 54.0±24.0 | 0.233 | 24.0±17.0 |
| ALT (IU/L) | 18.5±21.0 | 30.0±41.0 | 33.0±44.0 | 43.0±27.0 | 0.197 | 26.0±20.0 |
| Total bilirubin (mg/dL) | 0.30±0.18 | 0.39±0.29 | 0.55±0.21 | 0.52±0.20 | 0.071 | - |
| HDL cholesterol (mg/dL) | 45.5±30.0 | 44.5±24.0 | 34.0±24.0 | 42.0±16.0 | 0.795 | - |
| LDL cholesterol (mg/dL) | 58.5±71.0 | 101.0±91.0 | 71.0±44.0 | 67.0±50.0 | 0.413 | - |
| Triglyceride (mg/dL) | 123.0±170.0 | 146.0±135.0 | 81.0±92.0 | 81.0±149.0 | 0.978 | - |
| ESR (mm/hr) | 59.5±45.0 | 42.0±53.0 | 67.0±23.0 | 57.0±47.0 | 0.678 | - |
| CRP (mg/L) | 11.24±58.48 | 46.69±56.72 | 132.39±135.49 | 18.8±91.81 | 0.103 | - |
| Procalcitonin (ng/mL) | 0.32±0.73 | 0.09±0.09 | 0.16±0.16 | 0.07±0.04 | 0.068 | - |
| LDH (IU/L) | 559.5±100.0^a^ | 818.5±441.0^ab^ | 915.0±305.0^ab^ | 911.0±247.0^b^ | 0.046 | - |
| Ferritin (ng/mL) | 401.95±326.40 | 1236.50±1980.15 | 608.30±1233.05 | 702.10±520.80 | 0.677 | - |
| **Cytokine (pg/mL)** |  |  |  |  |  |  |
| IFN-γ | 3.11±8.93 | 0.00±11.13 | 0.00±13.42 | 0.43±10.02 | 0.831 | 0.00±0.00 |
| IL-10 | 2.55±10.42^ab^ | 6.60±4.66^a^ | 2.19±1.54^ab^ | 0.85±1.08^b^ | 0.011 | 0.12±0.56 |
| IL-2 | 0.00±0.00 | 0.00±6.72 | 0.00±4.61 | 0.00±0.33 | 0.620 | 0.00±0.00 |
| IL-6 | 16.11±18.16 | 9.40±64.35 | 2.10±5.69 | 6.67±21.06 | 0.107 | 0.00±0.00 |
| TNF-α | 8.21±11.53 | 1.84±2.40 | 3.72±1.53 | 5.63±10.39 | 0.062 | 1.19±3.88 |
| **Anti-SARS-CoV-2 S IgG (U/mL)** | 0.40±153.45^a^ | 0.40±0.10^a^ | 3.16±13.31^a^ | 4.31±34.27^a^ | 0.045 | - |
| Group 1, improved from mild COVID-19; Group 2, deterioration; Group 3, improved from moderate to mild severity; Group 4, improved from severe to mild severity.  WBC, white blood cell; ANC, absolute neutrophil count; MCV, mean corpuscular volume; MCH, mean corpuscular hemoglobin; MCHC, mean corpuscular hemoglobin concentration; BUN, blood urea nitrogen; HDL, high density lipoprotein; LDL, low density lipoprotein; AST, aspartate transaminase; ALT, alanine transaminase; GGT, gamma-glutamyl transferase; CRP, C-reactive protein; LDH, lactate dehydrogenase; IL, interleukin; TNF, tumor necrosis factor; IFN, interferon; N.A, not applicable.  *Superscripts* (^a,b^). For a particular variable, mode means with different superscript are significantly (*p* <0.05) different. Mode means with same superscripts are not significantly (*p* >0.05) different. When only one contrast is significant, one of the cells means has no superscript attached. The pair of cell means that is significant has different superscripts.  Continuous variables are shown as median ± interquartile range (IQR) and categorical variables as numbers (percentage). | | | | | | |

**Table S4. Differentially expressed proteins between mild case group (G1) and healthy control group**

| ***Increased proteins in the mild COVID-19 groups*** | | | | | | |
| --- | --- | --- | --- | --- | --- | --- |
| ***Uniprot ID*** | ***Protein*** | ***Uniprot description*** | | ***-log_10_***  ***p value*** | ***log_2_***  ***Fold change*** | ***Biological function & process*** |
| P00746 | CFAD | | **Complement factor D** | 2.752 | 4.14 | Innate immunity |
| P02750 | A2GL | | **Leucine-rich alpha-2-glycoprotein** | 3.557 | 3.57 | Response to bacterium |
| P02763 | A1AG1 | | **Alpha-1-acid glycoprotein 1** | 4.455 | 3.41 | Acute phase, Transport |
| A0A0C4DH30 | HV316 | | **Probable non-functional immunoglobulin heavy variable 3-16** | 3.533 | 3.22 | Immunity |
| P00739 | HPTR | | **Haptoglobin-related protein** | 2.961 | 2.52 | Acute inflammatory response |
| Q9NZP8 | C1RL | | **Complement C1r subcomponent-like protein** | 1.302 | 2.44 | Innate immunity |
| P22792 | CPN2 | | **Carboxypeptidase N subunit 2** | 2.945 | 2.41 | Protein stabilization |
| P43251 | BTD | | **Biotinidase** | 1.761 | 1.99 | Hydrolase |
| P01011 | AACT | | **Alpha-1-antichymotrypsin** | 4.189 | 1.94 | Acute phase |
| P04275 | VWF | | **von Willebrand factor** | 1.386 | 1.72 | Blood coagulation |
| P19652 | A1AG2 | | **Alpha-1-acid glycoprotein 2** | 1.623 | 1.70 | Acute phase, Transport |
| P00736 | C1R | | **Complement C1r subcomponent** | 4.483 | 1.67 | Complement pathway |
| Q9Y577 | TRI17 | | **E3 ubiquitin-protein ligase TRIM17** | 2.412 | 1.54 | Ubl conjugation pathway |
| P00738 | HPT | | **Haptoglobin** | 1.935 | 1.47 | Acute phase, Immunity |
| P02746 | C1QB | | **Complement C1q subcomponent subunit B** | 1.809 | 1.47 | Complement pathway |
| P06681 | CO2 | | **Complement C2** | 3.336 | 1.44 | Complement pathway |
| P10909 | CLUS | | **Clusterin** | 1.833 | 1.36 | Apoptosis, Complement pathway |
| P22352 | GPX3 | | **Glutathione peroxidase 3** | 1.904 | 1.23 | Hydrogen peroxide catabolic process |
| P01009 | A1AT | | **Alpha-1-antitrypsin** | 3.127 | 1.22 | Acute phase, Blood coagulation |
| P25311 | ZA2G | | **Zinc-alpha-2-glycoprotein** | 2.197 | 1.20 | Immune response |

| ***Decreased proteins in the mild COVID-19 groups*** | | | | | |
| --- | --- | --- | --- | --- | --- |
| ***Uniprot ID*** | ***Protein*** | ***Uniprot description*** | ***-log_10_***  ***p value*** | ***log_2_***  ***Fold change*** | ***Biological function & process*** |
| P02751 | FINC | **Fibronectin** | 3.188 | -3.62 | Heparin-binding |
| P27918 | PROP | **Properdin** | 2.486 | -2.04 | Complement pathway |
| P04070 | PROC | **Vitamin K-dependent protein C** | 1.868 | -1.95 | Serine protease |
| P00748 | FA12 | **Coagulation factor XII** | 3.148 | -1.89 | Serine protease |
| P02766 | TTHY | **Transthyretin** | 2.740 | -1.70 | Thyroid hormone |
| O95445 | APOM | **Apolipoprotein M** | 1.912 | -1.61 | Lipid transport |
| P08603 | CFAH | **Complement factor H** | 3.027 | -1.24 | Complement pathway, Host-virus interaction |
| P02745 | C1QA | **Complement C1q subcomponent subunit A** | 1.756 | -1.21 | Complement pathway, Host virus interaction |
| P01023 | A2MG | **Alpha-2-macroglobulin** | 2.100 | -1.04 | Serine protease inhibitor |

**Table S5. Differentially expressed proteins between deterioration (G2) and improved patient groups (G3 and G4)**

| ***Increased proteins in the improved groups*** | | | | | |  | |
| --- | --- | --- | --- | --- | --- | --- | --- |
| ***Uniprot ID*** | ***Protein*** | ***Uniprot description*** | ***-log_10_***  ***p value*** | ***log_2_***  ***Fold change*** | ***Biological function***  ***& process*** | | ***Patient***  ***group*** |
| P04070 | PROC | Vitamin K-dependent  protein C | 3.242 | 1.52 | Serine protease | | G2 vs. G3 |
| P01023 | A2MG | Alpha-2-macroglobulin | 1.834 | 1.12 | Serine protease inhibitor | | G2 vs. G3 |
| P08603 | CFAH | Complement factor H | 1.705 | 1.11 | Complement pathway | | G2 vs. G3 |
| P06276 | CHLE | Cholinesterase | 1.359 | 1.51 | Serine esterase | | G2 vs. G4 |
| P04003 | C4BPA | C4b-binding protein alpha | 1.788 | 1.09 | Complement pathway | | G2 vs. G4 |
| P02766 | TTHY | Transthyretin | 1.308 | 1.04 | Transport | | G2 vs. G4 |

| ***Decreased proteins in the improved groups*** | | | | | |  |
| --- | --- | --- | --- | --- | --- | --- |
| ***Uniprot ID*** | ***Protein*** | ***Uniprot description*** | ***-log_10_***  ***p value*** | ***log_2_***  ***Fold change*** | ***Biological function***  ***& process*** | ***Patient***  ***group*** |
| P06727 | APOA4 | Apolipoprotein A-IV | 1.636 | -3.40 | Lipid transport | G2 vs. G3 |
| P03952 | KLKB1 | Plasma kallikrein | 1.534 | -2.36 | Fibrinolysis | G2 vs. G3 |
| A0A0C4DH30 | HV316 | Probable non-functional immunoglobulin heavy variable 3-16 | 1.439 | -1.92 | Immunity | G2 vs. G3 |
| P10909 | CLUS | Clusterin | 3.098 | -1.67 | Complement pathway | G2 vs. G3 |
| P27169 | PON1 | Serum paraoxonase/arylesterase 1 | 1.301 | -1.33 | Hydrolase | G2 vs. G3 |
| P06681 | CO2 | Complement C2 | 1.329 | -1.22 | Complement pathway | G2 vs. G3 |
| Q9UGM5 | FETUB | Fetuin-B | 3.104 | -2.54 | Fertilization | G2 vs. G4 |
| P06681 | CO2 | Complement C2 | 1.680 | -1.33 | Complement pathway | G2 vs. G4 |
| P29622 | KAIN | Kallistatin | 1.619 | -1.22 | Serine protease inhibitor | G2 vs. G4 |

**Section 5: References**

1. Lee, S.J., et al., *Identification of Nucleolin as a Novel AEG-1-Interacting Protein in Breast Cancer via Interactome Profiling.* Cancers (Basel), 2021. **13**(11).

2. Lee, S.Y., et al., *Serum proteomics of severe fever with thrombocytopenia syndrome patients.* Clin Proteomics, 2022. **19**(1): p. 32.

3. Choi, K.M., et al., *Postmortem proteomics to discover biomarkers for forensic PMI estimation.* Int J Legal Med, 2019. **133**(3): p. 899-908.

4. Chawade, A., E. Alexandersson, and F. Levander, *Normalyzer: a tool for rapid evaluation of normalization methods for omics data sets.* J Proteome Res, 2014. **13**(6): p. 3114-20.

5. Java, A., et al., *The complement system in COVID-19: friend and foe?* JCI Insight, 2020. **5**(15).

6. Afzali, B., et al., *The state of complement in COVID-19.* Nat Rev Immunol, 2022. **22**(2): p. 77-84.

7. Yu, J., et al., *Complement dysregulation is associated with severe COVID-19 illness.* Haematologica, 2022. **107**(5): p. 1095-1105.

8. Rajamanickam, A., et al., *Levels of Complement Components in Children With Acute COVID-19 or Multisystem Inflammatory Syndrome.* JAMA Netw Open, 2023. **6**(3): p. e231713.

9. Ma, L., et al., *Increased complement activation is a distinctive feature of severe SARS-CoV-2 infection.* Sci Immunol, 2021. **6**(59).

10. Holter, J.C., et al., *Systemic complement activation is associated with respiratory failure in COVID-19 hospitalized patients.* Proc Natl Acad Sci U S A, 2020. **117**(40): p. 25018-25025.

11. AGNELLO, V., *Complement deficiency states.* Medicine, 1978. **57**(1): p. 1-24.

12. Blom, A.M., B.O. Villoutreix, and B. Dahlback, *Complement inhibitor C4b-binding protein-friend or foe in the innate immune system?* Mol Immunol, 2004. **40**(18): p. 1333-46.

13. Zipfel, P.F. and C. Skerka, *Complement regulators and inhibitory proteins.* Nat Rev Immunol, 2009. **9**(10): p. 729-40.

14. Kaplan, A.P. and B. Ghebrehiwet, *The plasma bradykinin-forming pathways and its interrelationships with complement.* Molecular immunology, 2010. **47**(13): p. 2161-2169.

15. Kolte, D. and Z. Shariat-Madar, *Plasma kallikrein inhibitors in cardiovascular disease: an innovative therapeutic approach.* Cardiology in Review, 2016. **24**(3): p. 99-109.

16. Esmon, C.T., *The roles of protein C and thrombomodulin in the regulation of blood coagulation.* Journal of Biological Chemistry, 1989. **264**(9): p. 4743-4746.

17. Shu, T., et al., *Plasma proteomics identify biomarkers and pathogenesis of COVID-19.* Immunity, 2020. **53**(5): p. 1108-1122. e5.

18. Feistritzer, C. and M. Riewald, *Endothelial barrier protection by activated protein C through PAR1-dependent sphingosine 1–phosphate receptor-1 crossactivation.* Blood, 2005. **105**(8): p. 3178-3184.

19. Imber, M. and S. Pizzo, *Clearance and binding of two electrophoretic “fast” forms of human alpha 2-macroglobulin.* Journal of Biological Chemistry, 1981. **256**(15): p. 8134-8139.
